# Commentary: How Long Do We Need to Follow-Up Our Hernia Patients to Find the Real Recurrence Rate?

**DOI:** 10.3389/fsurg.2015.00050

**Published:** 2015-09-23

**Authors:** Affan Umer, Scott Ellner

**Affiliations:** ^1^Department of Surgery, Saint Francis Hospital and Medical Center, University of Connecticut Health Center, Hartford, CT, USA

**Keywords:** hernia registry, inguinal hernia repair, incisional hernia repair, hernia repair mesh, hernia recurrence, hernia recurrence risk factors

It was a pleasure to go through the manuscript by Ferdinand Köckerling focusing on hernia recurrence in Frontiers in Surgery (1). We would like to applaud the authors on two fronts. We had very peripheral awareness of the Herniamed registry, but having read this paper, we believe that the registry is a brilliant initiative by the German surgical society. Such data sharing will most definitely help to evolve and standardize practices in hernia surgery across the board. Second, the results and recommendations of this study are novel in nature. The recurrence rates followed over such a long interval have important implications for managing follow-up care for hernia patients.

Recurrence and wound infection rates, postoperative neuralgia, hospital length of stay, and return to normal activity are important metrics that define the success of any incisional or inguinal hernia repair. Outcomes in hernia repair can not only be attributed to its inherent anatomy (size, location, severity) but also strongly influenced by the type of repair. There is sufficient literature that favors the superiority of mesh repair over suture repair (2–4). Similarly, Bassini repairs have higher recurrence rates when compared to the non-Bassini repairs (4). No significant difference in hernia recurrence has been found between open and laparoscopic techniques that utilized mesh (5), but differences still exist in return to normal activity and hospital length of stay. Another obstacle to the success of any herniorrhaphy is the experience of the surgeon. Aquina et al. found differences in surgeon experience contributing to the hernia recurrence rates, i.e., surgeons performing <25 cases annually were associated with a higher rate of recurrence [hazard ratio 1.23, 95% CI (1.11–1.36)] (6). Cumulative recurrence rates as presented by the author for inguinal and incisional hernias (1) are definitely useful, but we fear it might be an over-simplification. In light of the potential confounders, we would like to request the authors to present a subset analysis of recurrences over time compared between repairs with and without mesh, and also between herniorrhaphy done by high volume and low volume surgeons from their study cohort.

Our hypothesis is that a suture repair or any hernia repair in the hands of an inexperienced surgeon has a greater potential of being technically inadequate, and might require a close interval follow-up. On the contrary, a superior mesh repair done by an experienced surgeon could likely require a longer interval follow-up. In a subset analysis, Burger et al. (7) found that 67% of their incisional hernias recurred within 10 years in their suture repair group compared to 17% in the mesh group. Our concern is that in addition to the type of hernia, the type of repair should significantly influence recurrence patterns as well.

Nicolas Jean Marjolin stated back in 1828 that surgery has reached such a level of improvement that nothing further can be expected. That judgment may be far from the truth, but unfortunately the paragon of surgical precision still eludes us in this day and age. Inguinal herniorrhaphy is one of the most commonly performed general surgical procedures but it can still be technically challenging. Continuous training is essential to achieve proficiency. Part of the reason why hernia recurrences continue to plague us is because we continue to employ redundant techniques. As health care delivery systems become increasingly patient centered, patient outcomes, which in turn reflect on the quality of care delivered, will drive health care economics. This provides strong incentive to move forward with best care practices supported by evidence-based medicine guidelines in hernia surgery. The findings of the Herniamed registry are definitely a step in the right direction. In the future, such registries can potentially help us generate predictive models for complications, which include recurrence, and tackle them in a timely and more effective manner, if not completely avoid them altogether.

## Conflict of Interest Statement

The authors declare that the research was conducted in the absence of any commercial or financial relationships that could be construed as a potential conflict of interest.
